# Peripartal rumen-protected methionine supplementation to higher energy diets elicits positive effects on blood neutrophil gene networks, performance and liver lipid content in dairy cows

**DOI:** 10.1186/s40104-016-0077-9

**Published:** 2016-03-09

**Authors:** Cong Li, Fernanda Batistel, Johan Samir Osorio, James K. Drackley, Daniel Luchini, Juan J. Loor

**Affiliations:** Key Laboratory of Animal Genetics and Breeding of Ministry of Agriculture, National Engineering Laboratory of Animal Breeding, College of Animal Science and Technology, China Agricultural University, Beijing, 100193 China; Department of Animal Sciences and Division of Nutritional Sciences, University of Illinois, Urbana, IL 61801 USA; Department of Animal and Rangeland Sciences, Oregon State University, Corvallis, 97331 OR USA; Ruminant Technical Services, ADISSEO NA, Alpharetta, 30022 GA USA

**Keywords:** Blood neutrophil, Gene expression, Methionine

## Abstract

**Background:**

Main objectives were to determine to what extent Smartamine M (SM) supplementation to a prepartal higher-energy diet could alter neutrophil (PMN) and liver tissue immunometabolic biomarkers, and whether those responses were comparable to those in cows fed a prepartal lower-energy diet (CON).

**Results:**

Twenty-eight multiparous Holstein cows were fed CON (NE_L_ = 1.24 Mcal/kg DM) during d −50 to d −22 relative to calving. From d −21 to calving, cows were randomly assigned to a higher-energy diet (OVE, *n* = 9; NE_L_ = 1.54 Mcal/kg DM), OVE plus SM (OVE + SM, *n* = 10; SM = 0.07 % of DM) or remained on CON (*n* = 9). All cows received the same basal lactation diet (NE_L_ = 1.75 Mcal/kg DM). Supplementation of SM (OVE + SM) continued until 30 d postpartum. Liver biopsies were harvested at d −10, 7, and 21 relative to parturition. Blood PMN isolated at −10, 3, and 21 d relative to calving was used to evaluate gene expression. As expected, OVE increased liver lipid content postpartum; however, cows fed OVE + SM or CON had lower concentrations than OVE. Compared with OVE, cows in CON and OVE + SM had greater DMI postpartum and milk production. Furthermore, cows fed OVE + SM had the greatest milk protein and fat percentage and lowest milk SCC despite having intermediate PMN phagocytic capacity. Adaptations in PMN gene expression in OVE + SM cows associated with the lower SCC were gradual increases from −10 to 21 d in genes that facilitate migration into inflammatory sites (*SELL*, *ITGAM*), enzymes essential for reducing reactive oxygen metabolites (*SOD1*, *SOD2*), and a transcription factor(s) required for controlling PMN development (*RXRA*). The greater expression of *TLR4* on d 3, key for activation of innate immunity due to inflammation, in OVE compared with CON cows suggests a more pronounced inflammatory state. Feeding OVE + SM dampened the upregulation of *TLR4*, despite the fact that these cows had similar expression of the pro-inflammatory genes *NFKB1* and *TNF* as OVE. Cows in CON had lower overall expression of these inflammation-related genes and *GSR*, which generates reduced glutathione, an important cellular antioxidant.

**Conclusions:**

Although CON cows appeared to have a less stressful transition into lactation, SM supplementation was effective in alleviating negative effects of energy-overfeeding. As such, SM was beneficial in terms of production and appeared to boost the response of PMN in a way that improved overall cow health.

**Electronic supplementary material:**

The online version of this article (doi:10.1186/s40104-016-0077-9) contains supplementary material, which is available to authorized users.

## Background

Cows around calving time experience a depression on immune function partially due to the marked negative energy balance (NEB), which results when cows cannot ingest enough nutrients to support dietary requirements for milk production. During this time, methionine (Met) as one of the first limiting AA in dairy cows may be in limited supply. Research has demonstrated that Met plays a key role in milk protein synthesis, hepatic lipid metabolism, and immune function [1–3].

The decreased immune function during the peripartal period is partly responsible for the high incidence of infections [4, 5]. An effective immune response relies upon the efficient activation of polymorphonuclear neutrophils (PMN) [6]. PMN account for 25 % of leukocytes in bovine peripheral blood of healthy animals [7] and form the first line of cellular defense against invading pathogens [8].

Controlling prepartal energy intake has been associated not only with optimized hepatic lipid metabolism [9, 10] but also with a reduced inflammatory response after calving [9]. In contrast, energy-overfed cows often have greater hepatic lipid accumulation [11–13] increasing the risk of metabolic disorders during the peripartal period. Earlier studies have reported that over-feeding energy diets during the close-up period leads to a striking increase in serum NEFA and BHBA postcalving, both of which likely affect the immune response [14, 15]. We have recently observed that over-feeding energy during the dry period upregulated the expression of genes associated with the proinflammatory response such as *NFKB1*, *TLR2*, *RXRA*, and *PLA2G4A* [6].

Rumen-protected Met in the form of Smartamine M (SM; Adisseo NA, Alpharetta, GA, USA) is effective in providing extra metabolizable Met to balance peripartal diets, which in turn helps to optimize DMI, milk production, and improve whole blood phagocytosis capacity [3]. Our hypothesis was that SM during the peripartal period alleviates the negative effects of a prepartal higher-energy diet on PMN function as well as blood and liver tissue immunometabolic biomarkers, which are ultimately reflected in an impaired postpartal performance. Furthermore, it was hypothesized that beneficial effects of SM would result in responses comparable to those detected in cows fed a prepartal lower-energy diet. The hypothesis was addressed by measuring gene expression in PMN, biomarkers in blood and liver tissue, and performance.

## Methods

### Animals, experimental design, and animal management

Animal handling procedures were performed in accordance with protocols approved by the University of Illinois Institutional Animal Care and Use Committee. Complete details of the experimental design have been reported previously [3, 13]. Although published separately, all dietary treatments were run concurrently. Briefly, a subset of cows from a group of 65 that remained healthy throughout the study with the most complete set of PMN samples (d −10, 3, and 21) during the transition period were selected. All cows in the experiment were fed a lower-energy diet (CON; NE_L_ = 1.24 Mcal/kg DM; no Met supplementation) for ad libitum intake during the far-off dry period (i.e., d −50 to d −21). During the close-up period (i.e., d −21 d to calving), cows were randomly assigned either to a higher-energy diet (OVE; NE_L_ = 1.54 Mcal/kg DM), OVE plus Smartamine M (OVE + SM; Adisseo NA) or remained on CON (Table 1). The same basal lactation diet (NE_L_ = 1.75 Mcal/kg DM) was fed to all cows postpartum until 30 DIM. The number of cows used in the present study was 9, 10, and 9 in OVE, OVE + SM, and CON. The SM (0.07 % of DM) was top-dressed during the entire experiment over the OVE or lactation diet from -21 DIM through 30 DIM. Consecutive morning, midday, and evening milk samples were harvested until 30 DIM. Composite milk samples were prepared in proportion to milk yield at each milking, preserved (800 Broad Spectrum Microtabs II; D & F Control Systems Inc., Sab Ramon, CA), and analyzed for contents of fat, protein, lactose, and SCC (Dairy Lab Services, Dubuque, IL). The SCC data were log10 transformed prior to statistical analysis. Performance data from all cows in CON and OVE have been published previously by Ji et al. (2012), and data from all cows in OVE and OVE + SM by Osorio et al. [3]. Therefore, in order to combine the performance data for CON, OVE, and OVE + SM they were re-analyzed using only cows from which PMN were isolated.

**Table 1:** Ingredient and chemical composition of diets

	Close-up period^a^			Lactation^b^
Item	CON	OVE	OVE + SM	
Ingredient, % of DM				
Alfalfa silage	12.00	8.20	8.20	5.00
Alfalfa hay	—	3.50	3.50	4.00
Corn silage	33.00	35.90	35.90	33.00
Wheat straw	36.00	15.40	15.40	4.00
Cottonseed	—	—	—	3.50
Wet brewers grains	—	6.00	6.00	10.00
Ground shelled corn	4.00	13.00	13.00	22.20
Soy hulls	2.00	4.00	4.00	4.00
Soybean meal, 48 % CP	7.94	3.10	3.10	3.30
Expeller soybean meal^c^	—	2.00	2.00	6.20
SoyChlor^d^	0.15	3.80	3.80	—
Blood meal, 85 % CP	1.00	1.00	1.00	0.30
Smartamine M^e^	—	—	0.07	—
Urea	0.45	0.30	0.30	0.14
Rumen-inert fat^f^	—	—	—	1.00
Limestone	1.30	1.30	1.30	1.18
Salt (plain)	0.32	0.30	0.30	0.27
Dicalcium phosphate	0.12	0.18	0.18	0.27
Magnesium oxide	0.21	0.08	0.08	0.14
Magnesium sulfate	0.91	0.97	0.97	—
Sodium bicarbonate	—	—	—	0.75
Potassium carbonate	—	—	—	0.10
Calcium sulfate	—	—	—	0.10
Mineral-vitamin mix^g^	0.20	0.20	0.20	0.20
Vitamin A^h^	0.015	0.015	0.015	—
Vitamin D^i^	0.025	0.025	0.025	—
Vitamin E^j^	0.38	0.38	0.38	—
Biotin	—	0.35	0.35	0.35
DM^k^, %	46.6 ± 0.8	45.2 ± 0.8	45.2 ± 0.8	45.2 ± 1.5
Chemical analysis, %				
NE_L_, Mcal/kg	1.24	1.47	1.47	1.65
CP, % of DM	14.6	15.6	15.6	16.3
ADF, % of DM	36.2	30.2	30.2	24.1
NDF, % of DM	52.7	44.7	44.7	37.9

^a^The control diet (CON) was fed to all cows during the far-off dry period (−50 to −21 d relative to expected calving). During the close-up period (−21 d to calving) cows were assigned either to a higher-energy diet (OVE), OVE plus Smartamine M (OVE + SM) or continuously fed the CON diet

^b^All cows received the same lactation diet; however, Smartamine M (0.07 % of DMI) supplementation to the OVE + SM diet continued until 30 DIM

^c^SoyPLUS (West Central Soy, Ralston, IA, USA)

^d^SoyChlor (West Central Soy, Ralston, IA, USA)

^e^Smartamine M (Adisseo NA, Alpharetta, GA, USA)

^f^Energy Booster 100 (MSC, Carpentersville, IL, USA)

^g^Contained a minimum of 5 % Mg, 10 % S, 7.5 % K, 2.0 % Fe, 3.0 % Zn, 3.0 % Mn, 5000 mg of Cu/kg, 250 mg of I/kg, 40 mg of Co/kg, 150 mg of Se/kg, 2200 kIU of vitamin A/kg, 660 kIU of vitamin D3/kg, and 7700 IU of vitamin E/kg

^h^Contained 30,000 kIU/kg

^i^Contained 5009 kIU/kg

^j^Contained 44,000 IU/kg

^k^Means ± SD

### Blood metabolites and liver composition

Blood was sampled from the coccygeal vein at d −21, −10, 7, 14 and 21 relative to parturition. Samples were collected into evacuated serum tubes (BD Vacutainer; BD and Co., Franklin Lakes, NJ) containing either clot activator or lithium heparin for serum and plasma, respectively. After blood collection, tubes with lithium heparin were placed on ice and tubes with clot activator were kept at 21 °C until centrifugation (~30 min). Serum and plasma were obtained by centrifugation at 1900 × g for 15 min at 4 °C. Aliquots of serum and plasma were frozen (−20 °C) until further analysis. Measurements of NEFA, BHBA and glucose were performed using commercial kits in an autoanalyzer at the University of Illinois Veterinary Diagnostic Laboratory (Urbana). Insulin concentration was quantified using a commercial bovine insulin ELISA kit (catalog no. 10-1201-01; Mercodia AB, Uppsala, Sweden). The concentration of very-low-density lipoproteins (VLDL) was determined using a high-density lipoprotein and low-density lipoprotein (LDL)/VLDL cholesterol quantification kit (catalog no. K613-100; BioVision Inc., Mountain View, CA).

Liver biopsies were harvested at d −10, 7, and 21 relative to parturition from cows under local anesthesia using the same procedures as described previously (Osorio et al., 2013). Liver was frozen immediately in liquid nitrogen and stored until further analysis for concentration of total lipid [16] and triacylglycerol (TAG) [17, 18].

### PMN isolation

Neutrophils were isolated based on procedures described by Moyes et al. [19] with modifications. Briefly, blood (~120 mL) was sampled from the coccygeal vein before morning feeding at −10, 3, and 21 d in ACD Vacutainer tubes and mixed well by inversion and placed on ice until isolation. Samples were centrifuged at 600 × *g* for 30 min at 4 °C. The plasma, buffy coat, and approximately one-third of the red blood cells were discarded. The remaining sample was poured into a 50-mL conical tube (Fisher Scientific, Pittsburgh, PA). Twenty-five milliliters of deionized water at 4 °C was added to lyse red blood cells, followed by addition of 5 mL of 5 × PBS at 4 °C to restore an iso-osmotic environment. Samples were centrifuged at 200 × *g* for 10 min at 4 °C and the supernatants were decanted. The pellet was washed with 10 mL of 1 × PBS and centrifuged for 5 min (200 × *g* at 4 °C) and supernatants were decanted. Eight milliliters of deionized water at 4 °C was added, followed by addition of 2 mL of 5 × PBS at 4 °C. Samples were centrifuged at 500 × *g* for 5 min at 4 °C and supernatant was decanted. Two subsequent washings using 10 mL of 1 × PBS at 4 °C were performed with samples centrifuged at 500 × *g* for 5 min at 4 °C and supernatant was decanted. Although no cell differential was performed, this protocol routinely results in >88 % of isolated cells as neutrophils [19–21]. Neutrophils were immediately homogenized in 2 mL of Trizol Reagent (Invitrogen, Carlsbad, CA) with 1 μL of liner acrylamide (Ambion Inc., Austin, TX) using a Polytron power homogenizer at maximum speed. The suspension was transferred equally into 2 RNA-free microcentrifuge tubes (2 mL; Fisher Scientific) and stored at −80 °C until further analysis.

### Whole blood phagocytosis

Details of the phagocytosis procedure were reported previously [3]. The phagocytic capacity of heparinized whole blood was determined using the Phagotest kit (Orpegen Pharma, Heidelberg, Germany) following the manufacturer’s instructions. In brief, 20 μL of bacteria *Escherichia coli* was added to 1 of 3 whole blood samples (100 μL; 1 control and 2 test samples) in test tubes (Falcon, Becton Dickinson, Franklin Lakes, NJ) and incubated for 10 min at 37 °C. The cells were resuspended in 200 μL of DNA-staining solution, and light-protected in an ice bath until analyzed by flow cytometry (LSR II, Becton Dickinson, San Jose, CA).

### RNA extraction, primer design and evaluation, and quantitative PCR

Specific details of RNA extraction from PMN, primer design and evaluation, cDNA synthesis, and quantitative reverse transcription PCR are presented in the Additional File. Briefly, RNA samples were extracted from PMN using Qiazol reagent combination with miRNeasy® Mini Kit (Cat. #217004, Qiagen). The quality of RNA evaluated by RNA integrity number (RIN) in the 2100 Bioanalyzer (Agilent Technologies Inc., Santa Clara, CA) was above 6.50. Based on relevant biological functions in PMN, 16 target genes selected in this study are involved in metabolism, inflammation, oxidative stress, and cellular receptors. The official symbol, name, and a short summary description of these genes are presented in Additional file 1: Table S1. Primers were designed via Primer Express 3.0.1 software (Applied Biosystems).

Quantitative PCR (qPCR) was performed by ABI Prism 7900 HT SDS instrument (Applied Biosystems). Details of primer sequences and amplicon size, primer product sequencing information, and qPCR performance are presented in Additional file 1: Table S2, S3, S4, and S5. We used three genes as internal controls (ICG), oxysterol-binding protein-like 2 (*OSBPL2*), golgin subfamily A, member 5 (*GOLGA5*), and single-strand-selective monofunctional uracil-DNA glycosylase 1 (*SMUG1*). These were previously confirmed as stably expressed for PMN gene expression [19]. The final gene expression data were normalized with the geometric mean of the 3 ICG.

### Statistical analysis

Gene expression data were normalized by logarithmic transformation prior to statistical analysis. Data were analyzed with the Proc MIXED procedure of SAS 9.4 (SAS Institute Inc., Cary, NC) using diet, time, and diet × time as fixed effects and cow as random effect. The exponential correlation covariance structure SP for repeated measures was used for analysis of gene expression and phagocytosis data with the following model:

$$ {y}_{ijk}=\upmu +{D}_i+{T}_j+D{T}_{ij}+{\alpha}_k+{e}_{ijk} $$

Where y_ijk_ is the dependent, continuous variable; μ is the general mean; D_i_ is the fixed effect of the diet (*i* = 1, 2, or 3, namely, CON, OVE or OVE + SM); T_j_ is the fixed effect of the time (*j* = 1, 2, or 3, namely, −10, 3, or 21 DIM); DT_ij_ is the fixed effect of the *i*th treatment by the *j*th time of the interaction; α_k_ is the random effect of the individual; e_ijk_ is the random residual. For data of DMI, SCC, milk yield, and milk composition, which were equally-spaced, an autoregressive 1 covariance structure was used while the exponential correlation covariance structure SP (POW) was used for unequally spaced data from liver composition and blood metabolites.

## Results

### Performance and phagocytosis

**Fig. 1:**
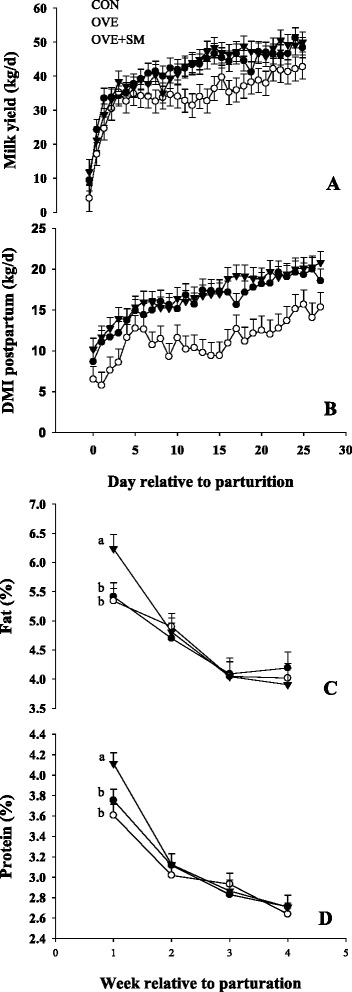
Postpartal milk yield (panel **a**), DMI (panel **b**), milk fat (panel **c**) and protein (panel **d**) content in Holstein cows fed a lower-energy control diet (CON), higher-energy diet (OVE) or OVE plus Smartamine M (OVE + SM) during the close-up period and through the first 30 d postpartum. ^a-b^Effect of diet (*P* < 0.10) at a specific time point

The complete set of milk yield, milk composition, and DMI data for the entire group of cows in CON, OVE, and OVE + SM have already been published by Osorio et al. [3] and Ji et al. [13]. Only performance data from cows used for PMN extraction were utilized in the present analysis. Results for peripartal performance including milk yield and components, SCC, ECM, DMI, and whole blood phagocytosis are presented in Table 2 and Fig. 1a-d. There was a D × T (*P* = 0.01) for DMI postpartum mainly due to a slower increment in DMI of OVE cows after 6 d postpartum. The latter was reflected in ca. 5.3 kg/d lower (*P* < 0.01) DMI in OVE than CON and OVE + SM, while no differences were observed between CON vs. OVE + SM. In contrast to DMI postpartum, prepartal DMI was not affected by diet or D × T.

**Table 2:** Effects of different treatments on production responses, somatic cell counts (SCC), whole blood phagocytosis, and blood and liver tissue biomarkers in Holstein cows fed a lower-energy diet (CON), higher-energy diet (OVE) or OVE plus Smartamine M (OVE + SM) during the close-up period and through the first 30 d postpartum

Item^d^	Treatment^a^			SEM^b^	*P*-value		
	CON	OVE	OVE + SM		Diet	Time	D × T^c^
Milk yield, kg/d	40.97^e^	34.04^f^	41.43^e^	2.59	0.07	<0.01	0.74
ECM, kg/d	44.82^e,f^	40.02^f^	46.90^e^	2.26	0.06	0.02	0.17
DMI, kg/d							
Prepartum	11.52	12.57	12.64	0.57	0.22	<0.01	0.76
Postpartum	16.19^e^	11.28^f^	16.94^e^	1.22	<0.01	<0.01	0.01
Feed efficiency	2.53^f^	3.01^e^	2.47^f^	0.15	0.03	<0.01	0.01
Milk fat, %	4.60	4.58	4.75	0.18	0.76	<0.01	0.09
Milk protein, %	3.10	3.05	3.20	0.09	0.44	<0.01	0.02
Log-transformed SCC	4.95^e^	4.92^e^	4.63^f^	0.10	0.06	<0.01	0.90
Whole blood phagocytosis, %	77.35^e^	41.81^g^	51.28^f^	3.38	<0.01	0.58	0.75
Blood biomarkers							
Insulin, μg/L^h^	−1.66^f^	−1.30^f^	−0.83^e^	0.19	0.01	<0.01	<0.01
NEFA, mEq/L^h^	−0.77	−0.96	−1.02	0.10	0.16	<0.01	<0.01
BHBA, mmol/L^h^	−0.39	−0.37	−0.52	0.08	0.30	<0.01	0.11
Glucose, mg/dL	57.8^e^	54.6^e,f^	53.5^f^	1.55	0.07	<0.01	0.54
VLDL, μg/μL	0.37^f^	0.43^e,f^	0.49^e^	0.04	0.01	<0.01	0.25
Liver composition, % wet wt							
Total lipid^h^	6.9	9.2	7.7	1.13	0.14	<0.01	0.37
TAG	2.47^f^	4.70^e^	3.40^e,f^	0.65	0.03	<0.01	0.15

^a^CON = lower-energy control energy (*n* = 9; 1.24 Mcal/kg DM); OVE = higher-energy (*n* = 9; 1.54 Mcal/kg DM); OVE + SM = higher-energy plus Smartamine M (*n* = 10; SM = 0.07 % of DM)

^b^Largest SEM is shown

^c^Interaction between diet and time

^d^ECM = energy corrected milk; VLDL = very-low density lipoproteins; TAG = triacylglycerol

^e-g^Mean values within a row with different superscripts were significantly different (*P* < 0.05)

^h^Log_2_-scale

There were D × T observed for milk protein (*P* = 0.02) and milk fat (*P* = 0.09), mainly attributed to greater (*P* < 0.01) concentration in OVE + SM than other treatments during the 1st wk of lactation. Milk yield was greater (*P* = 0.07) in CON and OVE + SM cows compared with OVE, while ECM (*P* = 0.06) was lower in OVE than OVE + SM but similar compared with CON. In addition, milk yield (*P* < 0.01) and ECM (*P* = 0.02) increased over time, while milk fat (*P* < 0.01) and milk protein (*P* < 0.01) decreased (Fig. 1a, c, d).

The SCC was lower in cows fed OVE + SM than CON and OVE (*P* < 0.04), while CON vs. OVE had similar SCC. Additionally, SCC declined (*P* < 0.01) over time after calving for all treatments. There was greater (*P* < 0.01) phagocytosis in whole blood of CON cows compared with OVE and OVE + SM, while OVE + SM cows had greater (*P* = 0.01) phagocytosis than OVE. In contrast to SCC, whole blood phagocytosis was not affected by time.

### Blood biomarkers and liver composition

**Fig. 2:**
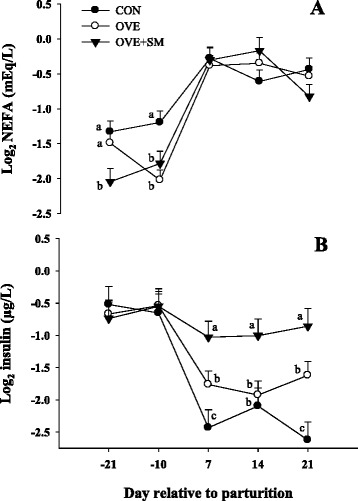
Non-esterified fatty acids (NEFA) (panel **a**) and insulin (panel **b**) concentration in Holstein cows fed a lower-energy control diet (CON), higher-energy diet (OVE) or OVE plus Smartamine M (OVE + SM) during the close-up period and through the first 30 d postpartum. ^a-c^Effect of diet (*P* < 0.10) at a specific time point

A D × T interaction was detected for insulin and NEFA concentration (*P* < 0.01; Table 2 and Fig. 2a, b). Feeding OVE + SM compared with OVE and CON led to greater (*P* < 0.02) insulin concentration after parturition, while cows in OVE compared with CON had greater (*P* < 0.01) insulin at d 7 and 21. Cows fed OVE + SM had lower (*P* = 0.03) NEFA at d -21 in comparison to OVE and CON, follow by lower NEFA in OVE and OVE + SM in comparison to CON at d −10 (*P* = 0.02) compared with CON, and at d −21. The concentration of BHBA was not affected (*P* > 0.05) by treatments.

Feeding OVE and OVE + SM compared with CON tended (*P* = 0.07) to decrease overall glucose concentration (Table 2). Although total lipid concentration in liver was not affected (*P* > 0.05) by treatments, the diet effect (*P* = 0.03) in concentration of TAG was reflected in lower TAG in cows fed CON (*P* = 0.01) and OVE + SM (*P* = 0.08) compared with OVE (Table 2). The concentration of VLDL was greater (*P* = 0.03) in OVE + SM fed cows compared with CON.

### Target gene expression

For most of the genes evaluated an interaction diet × time was observed, which based on the data was most likely associated with the different response over time between the CON and OVE + SM group.

### Met and glutathione metabolism

**Fig. 3:**
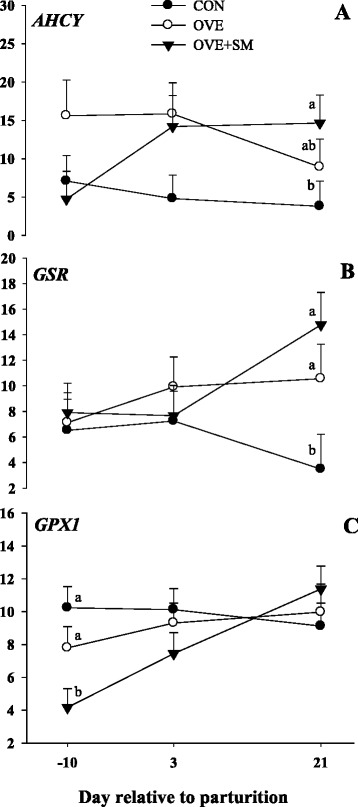
Expression of adenosylhomocysteinase (*AHCY*, panel **a**), glutathione reductase (*GSR*, panel **b**), and glutathione peroxidase 1 (*GPX1*, panel **c**) in PMN of Holstein cows fed a lower-energy control diet (CON), higher-energy diet (OVE) or OVE plus Smartamine M (OVE + SM) during the close-up period and through the first 30 d postpartum. These genes are related with methionine and glutathione metabolism. ^a-b^Effect of diet (*P* < 0.10) at a specific time point

A D × T interaction was observed for *GPX1* (*P* = 0.05), *AHCY* (*P* = 0.10) and *GSR* (*P* = 0.06; Table 3 and Fig. 3a-c). The expression of *AHCY* was lower (*P* = 0.02) in CON than OVE and OVE + SM, while expression was similar between OVE and OVE + SM cows. The expression of *GPX1* was lower in OVE + SM compared with CON (*P* = 0.01) and OVE (*P* = 0.01) at −10 d postpartum, and postpartal expression of *GPX1* was similar among treatments. At 21 d postpartum, CON cows had a lower expression of *GSR* compared with OVE (*P* = 0.07) and OVE + SM (*P* < 0.01), but *GSR* expression was similar between OVE and OVE + SM.

**Table 3:** Expression of target genes in PMN isolated on -10, +3, and 21 d around parturition in Holstein cows fed a lower-energy control diet (CON), a higher-energy diet (OVE) or OVE plus Smartamine M (OVE + SM) during the close-up period and through the first 30 d postpartum

Gene	Treatment^a^			SEM^b^	*P*-value		
	CON	OVE	OVE + SM		Diet	Time	D × T^c^
Met and glutathione metabolism							
* AHCY*	5.23^e^	13.47^d^	12.23^d^	2.47	0.02	0.67	0.10
* GSR*	5.76	9.21	10.12	2.02	0.28	0.34	0.06
* GPX1*	9.82	9.02	7.66	0.94	0.24	0.04	0.05
Inflammation							
* NFKB1*	7.80^e^	17.58^d^	19.24^d^	3.60	<0.01	0.80	0.98
* STAT3*	8.37	11.40	10.46	1.80	0.48	0.87	0.07
* TLR4*	−4.28^e^	−3.20^d,e^	−2.94^d^	0.57	0.07	0.96	0.06
* TNF*	−3.34^e^	5.91^d^	6.36^d^	2.05	<0.01	0.66	0.77
* LTA4H*	0.08	0.06	0.05	0.01	0.26	0.46	0.01
* RXRA*	11.32	12.94	13.39	1.59	0.61	0.11	0.03
Cellular receptors							
* SELL*	2.38	5.04	4.93	1.23	0.23	0.62	<0.01
* ITGAM*	5.04^e^	10.69^d^	10.37^d^	1.73	0.04	0.07	0.07
* TLN1*	12.53	9.77	10.08	1.11	0.18	0.24	0.07
* VCL*	−5.00^e^	−3.98^d^	−4.24^d^	0.26	0.02	<0.01	<0.01
Oxidative stress							
* SOD1*	7.09	9.93	10.31	1.98	0.34	0.36	0.10
* SOD2*	−0.68^e^	−0.83^e^	0.21^d^	0.32	0.06	0.04	0.08
* S100A8*	8.88	7.28	6.96	0.88	0.25	0.25	0.04

^a^CON = lower-energy control (*n* = 9; 1.24 Mcal/kg DM); OVE = higher-energy (*n* = 9; 1.54 Mcal/kg DM); OVE + SM = higher-energy plus Smartamine M (*n* = 10; SM = 0.07 % of DM)

^b^Largest SEM is shown

^c^ Interaction between diet and time

^d-e^Mean values within a row with different superscripts were significantly different (*P* < 0.05)

### Inflammation

**Fig. 4:**
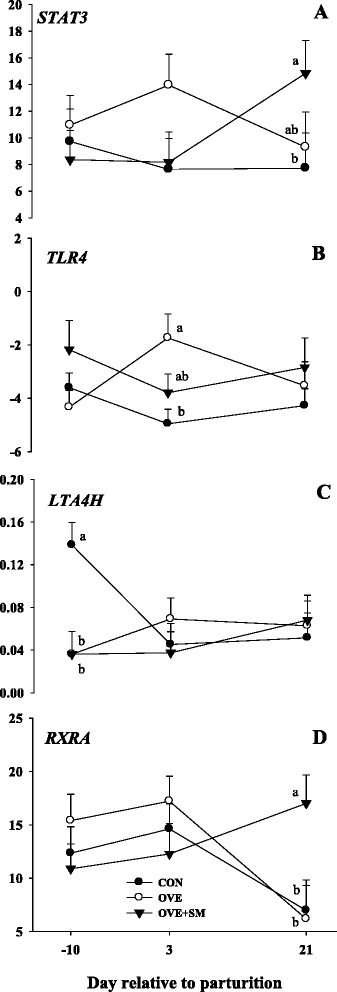
Expression of signal transducer and activator of transcription 3 (*STAT3*, panel **a**), Toll-like receptor 4 (*TLR4*, panel **b**), leukotriene A4 hydrolase (*LTA4H*, panel **c**), and retinoid X receptor, alpha (*RXRA*, panel **d**) in PMN of Holstein cows fed a lower-energy control diet (CON), higher-energy diet (OVE) or OVE plus Smartamine M (OVE + SM) during the close-up period and through the first 30 d postpartum. These genes are related with inflammation and gene transcription. ^a-b^Effect of diet (*P* < 0.10) at a specific time point

A D × T interaction was observed for *STAT3* (*P* = 0.07), *TLR4* (*P* = 0.06), *LTA4H* (*P* = 0.01) and *RXRA* (*P* = 0.03; Table 3 and Fig. 4a-d). A markedly lower (*P* < 0.01) expression of *LTA4H* at -10 d was observed in OVE + SM and OVE than CON fed cows. The mRNA expression of *TLR4* was greater (*P* = 0.01) in OVE than OVE + SM cows at 3 d, while *TLR4* expression in OVE + SM cows was similar to CON at the same time point. The expression of *STAT3* and *RXRA* was upregulated in OVE + SM cows than CON (*P* = 0.05). *STAT3* mRNA expression was similar between OVE and OVE + SM at 21 d postpartum, while *RXRA* mRNA expression was greater in OVE + SM than OVE at the same time point. The diet effect (*P* < 0.01) observed in *NFKB1* and *TNFA* was reflected in a greater mRNA expression of *NFKB1* (*P* < 0.01) and *TNFA* (*P* < 0.01) in OVE and OVE + SM cows compared with CON. The diet effect (*P* = 0.07) observed in *TLR4* was associated with a greater (*P* = 0.05) expression in OVE + SM than CON, while similar expression was observed between OVE + SM and OVE.

### Cellular receptors

**Fig. 5:**
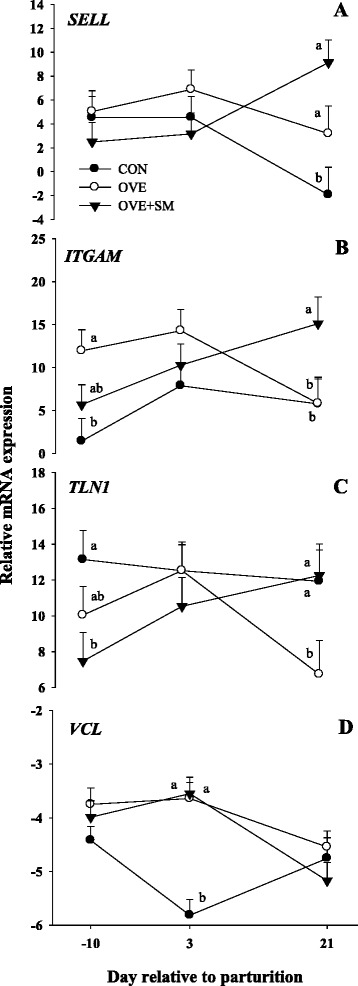
Expression of selectin L (*SELL*, panel **a**), integrin, alpha M (*ITGAM*, panel **b**), talin 1 (*TLN1*, panel **c**), and vinculin (*VCL*, panel **d**) in PMN of Holstein cows fed a lower-energy control diet (CON), higher-energy diet (OVE) or OVE plus Smartamine M (OVE + SM) during the close-up period and through the first 30 d postpartum. These genes are related with cellular receptors of PMN. ^a-b^Effect of diet (*P* < 0.10) at a specific time point

We observed a D × T interaction for the expression of *SELL* (*P* < 0.01), *ITGAM* (*P* = 0.07), *TLN1* (*P* = 0.07) and *VCL* (*P* < 0.01; Table 3 and Fig. 5a-d). The expression of *ITGAM* was greater (*P* = 0.01) in OVE than CON at −10 d, while expression in CON and OVE + SM was similar at the same time. The expression of *ITGAM* and *SELL* was greater in OVE + SM than CON (*P* = 0.01) and OVE (*P* < 0.01) cows at 21 d postpartum. The previous response in *ITGAM* was reflected in a diet effect (*P* = 0.04) were greater (*P* = 0.03) expression was observed in OVE and OVE + SM than CON. The expression of *TLN1* was markedly lower (*P* = 0.02) in OVE + SM than CON fed cows at -10 d, whereas expression in OVE + SM and OVE were similar. The *TLN1* was followed by lower (*P* = 0.05) expression in OVE than CON and OVE + SM at 21 d postpartum. Expression of *VCL* was drastically down-regulated in CON cows compared with OVE and OVE + SM (*P* < 0.01) cows at 3 d postpartum. Similarly to *ITGAM*, a diet effect (*P* = 0.02) was observed for *VCL* expression, where OVE and OVE + SM had greater expression than CON (*P* = 0.05).

### Oxidative stress

**Fig. 6:**
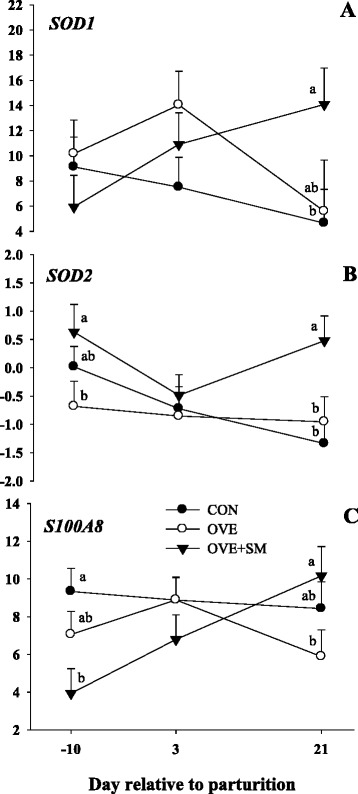
Expression of superoxide dismutase 1 (*SOD1*, panel **a**), superoxide dismutase 2 (*SOD2*, panel **b**), and S100 calcium binding protein A8 (*S100A8*, panel **c**) in PMN of Holstein cows fed a lower-energycontrol diet (CON), higher-energy diet (OVE) or OVE plus Smartamine M (OVE + SM) during the close-up period and through the first 30 d postpartum. These genes related with oxidative stress and inflammation. ^a-b^Effect of diet (*P* < 0.10) at a specific time point

A D × T interaction was observed for *SOD1* (*P* = 0.10), *SOD2* (*P* = 0.08), and *S100A8* (*P* = 0.04; Table 3 and Fig. 6a-c). Expression of *SOD2* was greater (*P* = 0.05) in OVE + SM than OVE cows at −10 d, while expressions in OVE and CON were similar. The expression of *SOD1* and *SOD2* was up-regulated in OVE + SM cows than CON (*P* = 0.02) and OVE (*P* = 0.09) at 21 d postpartum. The mRNA expression of *S100A8* was lower (*P* < 0.03) in OVE + SM than CON, while expression was similar between OVE and CON at −10 d. Similarly to prepartal expression of *SOD2*, the expression of *S100A8* was greater (*P* = 0.04) in OVE + SM than OVE at 21 d postpartum, while expression was similar between OVE and CON.

## Discussion

### Performance

Overfeeding dairy cows in the prepartum period typically increases NEFA concentration and liver TAG accumulation postpartum [12], which consequently can decrease milk yield, DMI, health status and reproductive performance [22]. Supplementing the diet with rumen-protected methyl donors (e.g. choline, Met) has sometimes resulted in lower liver TAG [23–25], due to an increase in phosphatidylcholine synthesis [26], which is a main constituent of VLDL [27]. Thus, the greater milk yield in OVE + SM than OVE could be attributed at least in part to a better health status of the liver which may have allowed cows to achieve a greater DMI. This hypothesis is partially supported by the lower liver TAG concentration and coupled with greater VLDL synthesis and export indicated by the greater blood VLDL concentration between OVE + SM vs CON but not OVE vs CON. The similar performance between CON and OVE + SM supports the idea that Met supplementation allowed cows to overcome the negative effects of the prepartal higher-energy diet. The greater ECM yield in OVE + SM cows compared with OVE was driven by the greater milk protein and milk fat response elicited by feeding SM [3].

### SCC and PMN phagocytosis

Phagocytosis is a key function of PMN, which are involved in host defense [28]. The Met supplied by the basal OVE diet along with tissue mobilization might not be sufficient to meet the demand by the immune system for sulphur amino acids, which is of central importance given that overfeeding dietary energy also could impair function of the immune system [6, 19]. It is well-established that metabolic products of Met metabolism, e.g. homocysteine, taurine and glutathione, play an important role in maintaining and supporting immune function [29]. The immunomodulatory properties of these compounds are underscored by the decrease in lymphocyte number and phagocytosis during taurine deprivation [30] as well as an increase in PMN adhesion when homocysteine concentration increased [30]. Furthermore, the antioxidant capacity of taurine and glutathione influences immune function by modulating the actions of reactive oxygen metabolites on transcription factor activation [31]. The whole blood phagocytic capacity detected in OVE + SM compared with OVE and CON provides evidence that enhancing Met supply could “boost” the immune system, hence, alleviating the negative effects of overfeeding energy in the dry period.

During mastitis, bacteria release toxins that activate macrophages and epithelial cells in the mammary gland to secrete cytokines that recruit PMN to the site of infection where they can serve as phagocytes [28]. The lower SCC in cows fed OVE + SM compared with CON and OVE might indicate that Met supplementation enhances immunity. Further research is needed to determine more precisely the effects (and mechanisms) of Met in cows that are more susceptible to mastitis risk.

### Gene expression

The mRNA expression of genes related to Met and glutathione metabolism, inflammation, and oxidative stress were evaluated to generate data on the possible mechanisms whereby Met elicits a response in PMN. The PMN function in dairy cows during the transition period is impaired in part due to high concentrations of NEFA and BHBA [32, 33]. Although in the present study NEFA and BHBA did not differ postpartum between treatments, the greater liver TAG accumulation in OVE than CON is indicative of a reduction in the capacity to export lipid out of the liver, also supported by the differences in blood VLDL concentration. Liver lipidosis clearly could impair cow performance. Research has demonstrated that increasing Met supply during the peripartal period increased hepatic expression of Met and glutathione metabolism-related genes, and decreased inflammation and oxidative stress [34]. However, to our knowledge, there are no published data reporting that Met supplementation has an effect on PMN from peripartal dairy cows.

### Methionine and glutathione metabolism

The enzyme S-adenosyl-L-homocysteine hydrolase (*AHCY*) is involved in the pathway from Met to homocysteine which is a precursor of glutathione [35]. Protection against the damaging effects of free radicals is carried out by GSR (glutathione reductase) and GPX1 (glutathione peroxidase), among others, which are enzymes related with glutathione metabolism [36]. Although it is possible that the increase in Met supply reaching the liver could have a positive effect on flux through the GSR and GPX1 pathways, the fact that *GSR* and *GPX1* did not differ indicates the existence of post-transcriptional control on both pathways.

### Inflammation

The genes *NFKB1* and *TNF* had the same pattern of response in OVE and OVE + SM cows. The greater *NFKB1* expression could be partly associated with the numerical increase of *STAT3* expression in those cows. It is well-established that the concentration of TNF-α, which stimulates the pro-inflammatory response, can be affected by several factors, e.g. tissue damage, pathogen invasion, and excessive fat deposition [37, 38]. The similar mRNA expression of *TNF* in OVE and OVE + SM indicates that the positive effect of Met supplementation may not be strictly related with PMN function, and that other mechanisms are more directly linked with the greater DMI in OVE + SM compared with OVE. The down-regulation of *RXRA* is essential for PMN development from granulocyte or monocyte progenitors [39], supporting other data indicating that retinoic acid deficiency led to an increase in neutrophil numbers in mice [40]. Although we did not measure retinoic acid or vitamin A concentrations in plasma or isolated neutrophils, it could be possible that the observed changes in *RXRA* were associated with the availability of these metabolites. Thus, as previously demonstrated in mice [40], the markedly greater expression of *RXRA* in OVE + SM cows at 21 d might have been associated with the stimulation of neutrophil differentiation. Although we are unaware of research studying the interaction of retinoic acids and Met in immune cells, there is evidence that exogenous retinoic acids alters Met catabolism in liver, i.e. enhances S-adenosylmethionie, S-adenosylhomocysteine, and taurine concentrations [41]. Thus, the observed change in *RXRA* in response to Met might have elicited a positive effect on the concentration of circulating neutrophils and their ability to control oxidative stress and inflammation.

### Cellular receptors

Neutrophils express a variety of adhesion molecules that are of fundamental importance in the acute inflammatory response by recognition of inflammatory sites, supporting adhesion, and transmigration across the endothelium as well as recognition and phagocytosis of opsonized microorganisms [42]. Among the four genes related with cellular receptors analyzed, *SELL* and *ITGAM* had a similar expression pattern in OVE + SM cows. Although homocysteine concentration was not measured, we speculate that feeding SM could have increased its concentration when compared with CON and OVE, and consequently, enhanced the ability for cell adhesion by the PMN as indicated by the greater *SELL* expression at d 21. Dudman et al. [30] reported that increasing homocysteine blood concentration from ≤10 μmol/L to ≥200 μmol/L increased neutrophil adhesion by ~50 %.

### Oxidative stress

Reactive oxygen metabolites (ROM) could serve as antimicrobial substances generated by the host defense mechanism to neutralize invading pathogens [43]. However, excessive production of ROM leads to loss of cell function, necrosis and apoptosis [44], and decreases dairy cow performance [45]. The imbalance between ROM production and the neutralizing capacity of antioxidant mechanisms is termed oxidative stress [46]. Antioxidant defenses are diverse and can be either synthesized in vivo or derived from the diet. The most efficient antioxidants are the enzymes SOD (SOD1, and SOD2), which can directly catalyze the reduction of ROM [47].

Hu et al. [48] reported that inhibition of *SOD2* caused accumulation of ROM. Thus, the upregulation of *SOD* isotypes in OVE + SM cows indicates that Met is linked to antioxidant mechanisms conferring protection against cell impairment from oxidative stress. Furthermore, several studies in non-ruminants have demonstrated direct protective effects of Met on oxidative stress [49–51] via the reaction of Met residues with ROM to form Met sulfoxide, hence, scavenging the reactive oxygen metabolites [49].

## Conclusions

The similar pro-inflammatory response in both overfed groups of cows with and without supplemental Met suggests that the mechanisms associated with the positive benefits of feeding Smartamine M are not only associated with the biology of the PMN. The temporal adaptations in PMN of genes related with migration, development and cellular antioxidants indicate that Smartamine M supplementation was effective in alleviating negative effects of prepartal energy-overfeeding. Furthermore, the similar DMI and milk yield of those cows compared with cows fed the lower-energy diet underscore the idea that Met helps overcome the limitations of overfeeding energy during the prepartal period.

## Additional file

Additional file 1: Table S1. Gene symbol, gene name, and description of the main biological function and biological process of the targets analyzed in PMN. **Table S2.** GeneBank accession number, hybridization position, sequence and amplicon size of primers used to analyze gene expression by qPCR. **Table S3.** Sequencing results of PCR products from primers of genes designed in this study. **Table S4.** Sequencing results of genes using BLASTN (http://www.ncbi.nlm.nih.gov) from NCBI against nucleotide collection (nr/nt) with total score. **Table S5.** qPCR performance among the genes measured in PMN. (DOCX 40 kb)
