# Supplementary material for: Peripartal rumen-protected methionine supplementation to higher energy diets elicits positive effects on blood neutrophil gene networks, performance and liver lipid content in dairy cows
Source: J Anim Sci Biotechnol. 2016 Mar 9;7:18. doi: 10.1186/s40104-016-0077-9 (PMC4784469; doi:10.1186/s40104-016-0077-9)
Supplement: Additional file 1: Table S1. — Gene symbol, gene name, and description of the main biological function and biological process of the targets analyzed in PMN. Table S2. GeneBank accession number, hybridization position, sequence and amplicon size of primers used to analyze gene expression by qPCR. Table S3. Sequencing results of PCR products from primers of genes designed in this study. Table S4. Sequencing results of genes using BLASTN (http://www.ncbi.nlm.nih.gov) from NCBI against nucleotide collection (nr/nt) with total score. Table S5. qPCR performance among the genes measured in PMN. (DOCX 40 kb) [file 40104_2016_77_MOESM1_ESM.docx]

**RNA extraction, Quality Assessment, and cDNA synthesis**

Total RNA was extracted from blood PMN using TRIZol regent combination with miRNeasy® Mini Kit (Cat. #217004, Qiagen). Isolated neutrophils were completely homogenized in 1 mL TRIZol reagent (Invitrogen, Carlsbad, CA) using Beadbeater twice for each 30 sec. Each tube was added into 200 μL Chloroform and put in room temperature 3 min after shaking vigorously for 15 sec. The upper phase was transferred into a new collection tube without disturbing the mid and lower phase after 12,000 g at 4℃ for 15 min centrifuge. Ethanol 100% of 750 μL was added and mixed well. All amount of liquid supernatants were pipetted into a miRNeasy mini spin column in a 2 mL collection tube, then followed manufacturer’s instructions of miRNeasy® Mini Kit. DNase I digestion mix (Cat. #79254, Qiagen) of 80 μL was added to each column to remove genomic DNA. Finally, 50 μL RNase free water were added to elute RNA and total RNA were obtained. The RNA concentration was measured with NanoDrop ND-1000 spectrophotometer (NanoDrop Technologies). The purity of RNA was assessed by ratio of optical density OD260/280, which were above 1.80 for all samples. The RNA integrity (RIN) was evaluated via electrophoretic analysis of 28S and 18S rRNA subunits using a 2100 Bioanalyzer (Agilent Technologies), and values were above 5.50 for all samples.

A portion of RNA was diluted to 100 ng/μL by adding DNase-RNase free water prior to cDNA synthesis. Complementary DNA was synthesized using 1 μL of 100 ng total RNA, 1 μL of Random Primers (Cat. #11034731001, Roche), and 9 μL of DNase/RNase-free water. The mixture was incubated at 65℃ for 5 min and kept on ice for 3 min. A total of 9 μL of master mix composed of 4 μL of 5X First-Strand Buffer (Cat. #18064-022, Invitrogen), 1 μL of Oligo dT18 (Operon Biotechnologies, Huntsville, AL), 2 μL of 10 mM dNTP mix (Cat. #18427-088, Invitrogen), 1.625 μL of DNase/RNase-free water, 0.25 μL (200U/μL) of Revert Aid Reverse Transcriptase (Cat. #EP0442, Thermo Scientific), and 0.125 μL (40U/μL) of RiboLock RNase Inhibitor (Cat. #EO0382, Thermo Scientific) were added. The reaction was performed in an Eppendorf Mastercycler Gradient following such temperature program: 25℃ for 5 min, 50℃ for 60 min, and 70℃ for 15 min. The cDNA was then diluted 1:4 with DNase/RNase-free water.

**Primer Design and Evaluation**

Primers were designed and evaluated as previously described (Bionaz and Loor, 2008). Briefly, primers were designed using Primer Express 3.0.1 (Applied Biosystems) with minimum amplicon size of 80 bp (amplicons of 100-120 bp were of superiority, if possible) and limited percentage of 3’ G + C. Major part of primer sets were designed to fall across exon-exon junctions. Then, primers were aligned against publicly available databases using BLASTN at NCBI and UCSC’s Cow (*Bos taurus*) Genome Browser Gateway to determine the compatibility of primers with already annotated sequence of the corresponding gene. Prior to qPCR, a 20 μL PCR reaction comprised of 8 μL dilute cDNA, 10 μL Power SYBR Green PCR Master Mix (Cat. #4367659, Applied Biosystems), 1 μL forward primer and 1 μL reverse primer was established to verify the primers. Of these, a universal reference cDNA amplified from all samples was utilized to ensure identification of desired genes. PCR product of 5 μL was run in a 2% agarose gel stained with SYBR Safe DNA Gel Stain (Cat. #S33102, Invitrogen), and the remaining 15 μL were cleaned with a QIAquick PCR Purification Kit (Cat. #28104, Qiagen) and sequenced at the Core DNA Sequencing Facility of the Roy J. Carver Biotechnology Center at the University of Illinois, Urbana. The sequencing product was confirmed through BLASTN at NCBI database. Only primers that did not present primer-dimer, a single band at the expected size in the gel, and had the right amplification product verified by sequencing were used for qPCR. The accuracy of a primer pair also was evaluated by the presence of a unique peak during the dissociation step at the end of qPCR. The biological functions of all target genes are presented in Supplemental Table S1. Supplemental Table S2 shows all designed primers in this study as well as all sequence information confirmed by BLASTN. Sequencing results for all genes are reported in Table S3.

**Quantitative PCR (qPCR)**

The qPCR was conducted in triplicate as described previously (Graugnard et al, 2009). Briefly, four microliters of diluted DNA (dilution 1:4) combined with 6 μL of mixture composed of 5 μL 1 × SYBR Green master mix (Cat. #4309155, Applied Biosystems), 0.4 μL each of 10 μM forward and reverse primers, and 0.2 μL of DNase/RNase-free water were added in a MicroAmp^TM^ Optical 384-Well Reaction Plate (Cat. #4309849, Applied Biosystems). A 6-point standard curve plus the nontemplate control (NTC) together with three replicates of each sample were run to detect the relative expression level. The reactions were conducted in ABI Prism 7900 HT SDS instrument (Applied Biosystems) following the conditions below: 2 min at 50℃, 10 min at 95℃, 40 cycles of 15 s at 95℃ (denaturation), and 1 min at 60℃ (annealing + extension). The presence of a single PCR product was verified by the dissociation protocol using incremental temperatures to 95℃ for 15 s, then 65℃ for 15 s. The threshold cycle (Ct) data were analyzed and transformed using the standard curve with the 7900 HT Sequence Detection Systems Software (version 2.2.1, Applied Biosystems). The final data were normalized with the geometric mean of the 3 ICGs, as reported previously (Moyes et al, 2010).

**Relative mRNA Abundance of Genes within PMN**

Efficiency of qPCR amplification for each gene was calculated using the standard curve method (Efficiency = 10^(-1/slope)^). Relative mRNA abundance among measured genes was calculated as previously reported (Bionaz and Loor, 2008), using the inverse of PCR efficiency raised to ∆Ct (gene abundance = 1/E^∆Ct^, where ∆Ct = Ct of tested gene – geometric mean Ct of 3 ICGs). Overall mRNA abundance for each gene among all samples of the same PMN was calculated using the median ∆Ct, and overall percentage of relative mRNA abundance was computed from the equation: 100 × mRNA abundance of each individual gene / sum of mRNA abundance of all the genes investigated. Supplemental Table 4 shows the qPCR performance among the genes measured in PMN.

**Additional file 1: Table S1.** Gene symbol, gene name, and description of the main biological function and biological process of the targets analyzed in PMN.

| Symbol | Name | Summary description from NCBI |
| --- | --- | --- |
| *GOLGA5* | Golgin A5 | Involved in maintaining Golgi structure. Stimulates the formation of Golgi stacks and ribbons. Involved in intra-Golgi retrograde transport |
| *GPX1* | Glutathione peroxidase | This gene encodes a member of the glutathione peroxidase family. Glutathione peroxidase functions in the detoxification of hydrogen peroxide, and is one of the most important antioxidant enzymes in humans |
| *GSR* | Glutathione reductase | This enzyme is a homodimeric flavoprotein. It is a central enzyme of cellular antioxidant defense, and reduces oxidized glutathione disulfide (GSSG) to the sulfhydryl form GSH, which is an important cellular antioxidant |
| *ITGAM* | Integrin, Alpha M | This gene encodes the integrin alpha M chain. Integrins are heterodimeric integral membrane proteins composed of an alpha chain and a beta chain. This I-domain containing alpha integrin combines with the beta 2 chain (ITGB2) to form a leukocyte-specific integrin referred to as macrophage receptor 1 ('Mac-1'), or inactivated-C3b (iC3b) receptor 3 ('CR3') |
| *LTA4H* | Leukotriene A4 Hydrolase | Leukotriene A4 hydrolase, (LTA4 hydrolase, LTA4H) is a zinc-dependent epoxide hydrolase which hydrolyzes LTA4 to form LTB4 during leukotriene biosynthesis. The enzyme is the only member of the leukotriene biosynthetic pathway which is mainly cytoplasmic |
| *NFKB1* | Nuclear Factor Of Kappa Light Polypeptide Gene Enhancer In B-Cells 1 | NF-kappa-B is a pleiotropic transcription factor present in almost all cell types and is the endpoint of a series of signal transduction events that are initiated by a vast array of stimuli related to many biological processes such as inflammation, immunity, differentiation, cell growth, tumorigenesis and apoptosis |
| *OSBPL2* | Oxysterol binding protein-like 2 | Binds phospholipids; exhibits strong binding to phosphatidic acid and weak binding to phosphatidylinositol 3-phosphate |
| *RXRA* | Retinoid X receptor, alpha | Receptor for retinoic acid. Retinoic acid receptors bind as heterodimers to their target response elements in response to their ligands, all-trans or 9-cis retinoic acid, and regulate gene expression in various biological processes |
| *S100A8* | S100 Calcium Binding Protein A8 | The protein encoded by this gene is a member of the S100 family of proteins containing 2 EF-hand calcium-binding motifs. S100 proteins are localized in the cytoplasm and/or nucleus of a wide range of cells, and involved in the regulation of a number of cellular processes such as cell cycle progression and differentiation |
| *SAHH* | Adenosylhomocysteinase | S-adenosylhomocysteine hydrolase catalyzes the reversible hydrolysis of S-adenosylhomocysteine (AdoHcy) to adenosine (Ado) and L-homocysteine (Hcy). Thus, it regulates the intracellular S-adenosylhomocysteine (SAH) concentration thought to be important for transmethylation reactions |
| *SELL* | Selectin L | This gene encodes a cell surface adhesion molecule that belongs to a family of adhesion/homing receptors. The encodedprotein contains a C-type lectin-like domain, a calcium-binding epidermal growth factor-like domain, and two short complement-like repeats |
| *SMUG1* | Single-strand-selective monofunctional uracil-DNA glycosylase 1 | This gene encodes a protein that participates in base excision repair by removing uracil from single- and double-stranded DNA |
| *SOD1* | Superoxide dismutase 1, cytosolic | The protein encoded by this gene binds copper and zinc ions and is one of two isozymes responsible for destroying free superoxide radicals in the body. The encoded isozyme is a soluble cytoplasmic protein, acting as a homodimer to convert naturally-occuring but harmful superoxide radicals to molecular oxygen and hydrogen peroxide |
| *SOD2* | Superoxide dismutase 2, mitochondrial | Destroys superoxide anion radicals which are normally produced within the cells and which are toxic to biological systems |
| *STAT3* | Signal Transducer And Activator Of Transcription 3 | Signal transducer and transcription activator that mediates cellular responses to interleukins |
| *TLN1* | Talin 1 | This gene encodes a cytoskeletal protein that is concentrated in areas of cell-substratum and cell-cell contacts. The encoded protein plays a significant role in the assembly of actin filaments and in spreading and migration of various cell types, including fibroblasts and osteoclasts |
| *TLR4* | Toll-like receptor 4 | Cooperates with LY96 and CD14 to mediate the innate immune response to bacterial lipopolysaccharide  (LPS). Acts via MYD88, TIRAP and TRAF6, leading to NF-kappa-B activation, cytokine secretion and the inflammatory response. Also involved in LPS-independent inflammatory responses triggered by Ni (2+). These responses require non-conserved histidines and are, therefore, species-specific |
| *TNF* | Tumor necrosis factor alpha | This gene encodes a multifunctional proinflammatory cytokine that belongs to the tumor necrosis factor (TNF) superfamily. This cytokine is mainly secreted by macrophages |
| *VCL* | Vinculin | Vinculin is a cytoskeletal protein associated with cell-cell and cell-matrix junctions, where it is thought to function as one of several interacting proteins involved in anchoring F-actin to the membrane |

**Additional file 1: Table S2.** GeneBank accession number, hybridization position, sequence and amplicon size of primers used to analyze gene expression by qPCR

| Accession # | Gene | Primers^1^ | Primers (5’-3’) | bp^2^ |
| --- | --- | --- | --- | --- |
| NM_001098007.1 | *GOLGA5* | F.1370  R.1472 | GAGCTACAGCAGCAAGTCAAAGTG  CTTTAGACTGGAGTATTCGAGTAGCT | 103 |
| NM_174076.3 | *GPX1* | F.325  R.445 | CCCCTGCAACCAGTTTGG  CTCGCACTTTTCGAAGAGCAT | 121 |
| NM_001114190.2 | *GSR* | F.1555  R.1654 | CGCTGAGAACCCAGAGACTTG  AAACGGAAAGTGGGAACAGTAAGTA | 100 |
| NM_001039957.1 | *ITGAM* | F.268  R.362 | GGCTTGTCTCTTGCATTTGCT  CCATTTGCATAGGTGTTCTCCTT | 95 |
| NM_001034280.1 | *LTA4H* | F.1042  R .1141 | ACATTTGTGGACGACTGTTTGGT  TGGGTCTCCCCAAAAGTCTTT | 100 |
| NM_001076409.1 | *NFKB1* | F.172  R.266 | TTCAACCGGAGATGCCACTAC ACACACGTAACGGAAACGAAATC | 95 |
| NM_001035020.2 | *OSBPL2* | F.196  R.295 | TGCCGTCACAGGCTTTGAC  CCATTACTTGCTGGTGTCCACAT | 100 |
| XM_881943.5 | *RXRA* | F.1215  R.1347 | TGTCCCCGATGAGCTTGAAG  GAGGCGTACTGCAAACACAAGT | 131 |
| NM_001113725.2 | *S100A8* | F.41  R.140 | CAAATCCTTGGACACCATGCT  GGCGTGGTAATTCCCTTTTTT | 100 |
| NM_001034315.1 | *SAHH* | F.887  R.995 | TGTCAGGAGGGCAACATCTTT  AGTGCCCAATGTTACACACAATG | 109 |
| NM_174182.1 | *SELL* | F.588  R.691 | CTCTGCTACACAGCTTCTTGTAAACC  CCGTAGTACCCCAAATCACAGTT | 104 |
| NM_001014958.1 | *SMUG1* | F.258  R.374 | CAGCTACGTGACCCGCTACTG  CGGACTACACTCACTTCACCAAAG | 117 |
| XM_005201085.1 | *SOD1* | F.268  R.368 | GGCTGTACCAGTGCAGGTCC  GCTGTCACATTGCCCAGGT | 101 |
| NM_201527.2 | *SOD2* | F.620  R.714 | TGTGGGAGCATGCTTATTACCTT  TGCAGTTACATTCTCCCAGTTGA | 95 |
| NM_001012671.2 | *STAT3* | F.3804  R.3913 | GGTAGCATGTGGGATGGTCTCT  GCATCCCTAGAAACTCTGGTCAA | 110 |
| XM_005210127.1 | *TLN1* | F.1108  R.1207 | TTCCTGCCCAAGGAGTATGTG  AGCGTACCTTGGCCTCAATCT | 100 |
| NM_174198.6 | *TLR4* | F.555  R.664 | TGCGTACAGGTTGTTCCTAACATT  TAGTTAAAGCTCAGGTCCAGCATCT | 110 |
| NM_173966.3 | *TNF* | F.367  R.480 | CCAGAGGGAAGAGCAGTCCC  TCGGCTACAACGTGGGCTAC | 114 |
| NM_001191370.1 | *VCL* | F.1778  R.1880 | CATCTCAGCTCCAAGACTCCTTAAA  TTGATGGGAGTCGTGGTATCAC | 103 |

^1^ Primer direction (F – forward; R – reverse) and hybridization position on the sequence.

^2^ Amplicon size in base pair (bp).

**Additional file 1: Table S3.** Sequencing results of PCR products from primers of genes designed in this study

| Gene | Sequence |
| --- | --- |
| *GOLGA5* | CCGATCCAGCTTGGACTCCTCTAGCAGGAATTAGCTGACTACAAGCAAAAAGCTACTCG  AATACTCCAGTCTAAAGGA |
| *GPX1* | GAACGAGAGATCCTGAATTGCCTGAAGTACGTCCGACCAGGCGGCGGGTTCGAGCCCA  ACTTTATGCTCTTCGAAAAGTGCGAG |
| *GSR* | GGCCAGCTGGACCAGTAGACCTTCGGGAAGGAACCAAATCATCACGTTTACTTACTGT  TCCCACTTTCCGTTT |
| *ITGAM* | CGACTCTGCGTCGTGCGTGTGCCCACAGGTCACCAAATTTGCAAGGAGAACACCTATG  CAAATGGAA |
| *LTA4H* | GAAGAGTCATTTCCGGCTCGTGGGCGGAGTGGAGAACTCCAGAATTCGATAAAGACT  TTTGGGGGAGACCCAACCCCTTT |
| *NFKB1* | CGATATCTTCGTGTCAAGCAAAAGTATTCGCAACACTGGAAGCACGAATGACAGATG  CCTGTATACGGGGCATCAGAAGGCCGTA |
| *OSBPL2* | CTTCCGGGAATTTCAGAGGCGAATCAGAGACTCACTGGCGTGATTCATGTGGACACC  AGCAAGTAATGGA |
| *RXRA* | ACAGCATTGAGGCAATGGAGCGCAGAGCGGGCAGGCGCAGCAGCAGCTTGGCGAAC  CTTCCTGGCTGCTCGGGGTACTTGTGTTTGCAGTACGCCTCA |
| *S100A8* | ATCTTTACCCTCTGACTTTGCTAATCGGATCTTCACAGAAAGCGGACACTTGGTGCTAG  TTACGATCAATCAGGATGGTGGAATTAACT |
| *SAHH* | CTGTACTGCATCATTCTTGGCCAGCACTTTGAACAGATGAAGGATGATGCCATTGTGTG  TAACATTGGGCACT |
| *SELL* | ACGGGCCATGGACAATGTGTGGAAGTCATCAATAATTACACCTGCGTGCCCGTGATTT  GGGGGGACCTCCGGAA |
| *SMUG1* | AGCAAGTGCTCTTCTTGGGCATGAACCCAGGACCCTTTGGCATGGCCCAGACGGGGGT  GCCCTTTGGTGAAGTGAGTGTAGTCCGA |
| *SOD1* | GTCCAAAAACCGGTGGGCCAAAAGATGAAGAGAGGCATGTTGGAGACCTGGGCAATG  TGACAGCT |
| *SOD2* | GCATGTTTGGCCGATTATCTGAGGCCATTTTGGAATGTGATCAACTGGGAGAATGTAA  CTGCAATAC |
| *STAT3* | GCATCCCTCTACGAGCACGGCTAGATGTGGTCGGCTACAGCCATCTTGTCTCAGTTGA  CCAGAGTTTCTAGGGATGCAA |
| *TLN1* | ACCCCGTCCGCAATTCCTTCTCTACGTGCAGGCACGAGATGCCTAGGAAATGGCTCCC  ATCCTGTCTCCTCTGCTAAAGGCC |
| *TLR4* | TGCGATGGAGCTGAATCTCTACAAATCCCCGACAACATCCCCATATCAACCAAGATGC  TGGACCTGAGCTTTAACTA |
| *TNF* | AGCCTCGTGGTTCAGAACACTCAGGTCCTCTTCTCAAGCCTCAAGTAACAAGCCGGTA  GCCCACGTTGTAGCCGA |
| *VCL* | GCAGTAGACCGGAGTCGGGAGGCAGTACCTCAGGAGGTGTCAGATGTTTTCAGTGAT  ACCACGACTCCCATCAACACTTT |

**Additional file 1: Table S4.** Sequencing results of genes using BLASTN (<http://www.ncbi.nlm.nih.gov>) from NCBI against nucleotide collection (nr/nt) with total score

| Gene | Best hit in NCBI | Score |
| --- | --- | --- |
| *GOLGA5* | Bos taurus golgin A5 (GOLGA5), mRNA | 120 |
| *GPX1* | Bos taurus glutathione peroxidase 1 (GPX1), mRNA | 145 |
| *GSR* | Bos taurus glutathione reductase (GSR), mRNA | 127 |
| *ITGAM* | Bos taurus integrin, alpha M (complement component 3 receptor 3 subunit) (ITGAM), mRNA | 71.6 |
| *LTA4H* | Bos taurus leukotriene A4 hydrolase (LTA4H), mRNA | 78.8 |
| *NFKB1* | Bos taurus nuclear factor of kappa light polypeptide gene enhancer in B-cells 1 (NFKB1), mRNA | 89.7 |
| *OSBPL2* | Bos taurus oxysterol binding protein-like 2 (OSBPL2), mRNA | 104 |
| *RXRA* | Bos taurus retinoid X receptor, alpha (RXRA), transcript variant 4, mRNA | 158 |
| *S100A8* | Bos taurus S100 calcium binding protein A8 (S100A8), mRNA | 51.8 |
| *SAHH* | Bos taurus adenosylhomocysteinase (AHCY), mRNA | 125 |
| *SELL* | Bos taurus selectin L (SELL), mRNA | 82.4 |
| *SMUG1* | Bos taurus single-strand-selective monofunctional uracil-DNA glycosylase 1 (SMUG1), mRNA | 154 |
| *SOD1* | Bos taurus superoxide dismutase 1, soluble (SOD1), mRNA | 105 |
| *SOD2* | Bos taurus superoxide dismutase 2, mitochondrial (SOD2), mRNA | 82.4 |
| *STAT3* | Bos taurus signal transducer and activator of transcription 3 (acute-phase response factor) (STAT3), mRNA | 107 |
| *TLN1* | Bos taurus talin 1 (TLN1), mRNA | 73.4 |
| *TLR4* | Bos taurus toll-like receptor 4 (TLR4), mRNA | 125 |
| *TNF* | Bos taurus tumor necrosis factor (TNF), mRNA | 116 |
| *VCL* | Bos taurus vinculin (VCL), mRNA | 89.7 |

**Additional file 1: Table S5.** qPCR performance among the genes measured in PMN

| Gene | Median Ct | Median ∆Ct^a^ | Slope | R^2b^ | Efficiency^c^ | Relative % mRNA abundance^d^ |
| --- | --- | --- | --- | --- | --- | --- |
| *GPX1* | 8.58 | -14.71 | -3.47 | 0.99 | 1.94 | 4.37 |
| *GSR* | 9.18 | -11.80 | -3.60 | 0.99 | 1.90 | 0.24 |
| *ITGAM* | 4.67 | -14.61 | -3.69 | 0.94 | 1.87 | 3.96 |
| *LTA4H* | 26.59 | 0.12 | -3.02 | 0.95 | 2.10 | 1.59E-06 |
| *NFKB1* | 6.68 | -14.23 | -3.19 | 0.97 | 2.06 | 2.72 |
| *RXRA* | 5.71 | -15.28 | -3.06 | 0.97 | 2.12 | 7.77 |
| *S100A8* | 6.69 | -16.86 | -3.46 | 0.99 | 1.94 | 37.60 |
| *SAHH* | 7.15 | -12.80 | -3.14 | 0.97 | 2.08 | 0.65 |
| *SELL* | 12.65 | -11.21 | -3.14 | 0.97 | 2.08 | 0.13 |
| *SOD1* | 9.35 | -11.78 | -3.39 | 0.94 | 1.97 | 0.23 |
| *SOD2* | 19.29 | -7.08 | -3.17 | 0.99 | 2.07 | 0.21E-02 |
| *STAT3* | 8.38 | -14.29 | -3.17 | 0.97 | 2.07 | 2.89 |
| *TLN1* | 5.69 | -16.91 | -3.37 | 0.96 | 1.98 | 39.42 |
| *TLR4* | 24.51 | -2.12 | -3.12 | 0.97 | 2.09 | 1.50E-05 |
| *TNF* | 27.20 | -8.54 | -4.75 | 0.96 | 1.62 | 0.01 |
| *VCL* | 25.71 | -0.83 | -3.05 | 0.99 | 2.06 | 4.10E-06 |

^a^ The median ∆Ct was calculated as [Ct gene – geometric mean of Ct internal controls] for each sample.

^b^ R^2^ = coefficient of determination of the standard curve.

^c^ Efficiency is calculated as [10^(-1 / Slope)^].

^d^ Detailed information about calculation of relative mRNA abundance is referred to supplemental materials and methods.
